# TOR as a Regulatory Target in *Rhipicephalus microplus* Embryogenesis

**DOI:** 10.3389/fphys.2019.00965

**Published:** 2019-07-31

**Authors:** Camila Waltero, Leonardo Araujo de Abreu, Thayná Alonso, Rodrigo Nunes-da-Fonseca, Itabajara da Silva Vaz, Carlos Logullo

**Affiliations:** ^1^Laboratório Integrado de Bioquímica Hatisaburo Masuda and Laboratório Integrado de Ciências Morfofuncionais, Instituto de Biodiversidade e Sustentabilidade NUPEM, Universidade Federal do Rio de Janeiro, Macaé, Brazil; ^2^Instituto Nacional de Ciência e Tecnologia em Entomologia Molecular (INCT-EM), Rio de Janeiro, Brazil; ^3^Centro de Biotecnologia, Faculdade de Veterinária, Universidade Federal do Rio Grande do Sul, Porto Alegre, Brazil

**Keywords:** target of rapamycin, embryogenesis, tick embryonic cells, BME26, *Rhipicephalus microplus*

## Abstract

Embryogenesis is a metabolically intensive process carried out under tightly controlled conditions. The insulin signaling pathway regulates glucose homeostasis and is essential for reproduction in metazoan model species. Three key targets are part of this signaling pathway: protein kinase B (PKB, or AKT), glycogen synthase kinase 3 (GSK-3), and target of rapamycin (TOR). While the role of AKT and GSK-3 has been investigated during tick embryonic development, the role of TOR remains unknown. In this study, TOR and two other downstream effectors, namely S6 kinase (S6K) and eukaryotic translation initiation factor 4E-binding protein 1 (4E-BP1), were investigated in *in vitro* studies using the tick embryonic cell line BME26. First, we show that exogenous insulin can stimulate TOR transcription. Second, TOR chemical inhibition led to a decrease in BME26 cell viability, loss of membrane integrity, and downregulation of S6K and 4E-BP1 transcription. Conversely, treating BME26 cells with chemical inhibitors of AKT or GSK-3 did not affect S6K and 4E-BP1 transcription, showing that TOR is specifically required to activate its downstream targets. To address the role of TOR in tick reproduction, *in vivo* studies were performed. Analysis of relative transcription during different stages of tick embryonic development showed different levels of transcription for TOR, and a maternal deposition of S6K and 4E-BP1 transcripts. Injection of TOR double-stranded RNA (dsRNA) into partially fed females led to a slight delay in oviposition, an atypical egg external morphology, decreased vitellin content in eggs, and decreased larval hatching. Taken together, our data show that the TOR signaling pathway is important for tick reproduction, that TOR acts as a regulatory target in *Rhipicephalus microplus* embryogenesis and represents a promising target for the development of compounds for tick control.

## Introduction

The cattle tick, *Rhipicephalus microplus*, is an obligate hematophagous ectoparasite of veterinary and economical relevance in tropical and subtropical regions, due to its role as animal disease vector for *Babesia* spp. and *Anaplasma* spp., and its impact on livestock production (Grisi et al., 2002; Guerrero et al., 2012; Parizi et al., 2012). Current methods for tick and tick-borne disease control depend heavily on the application of acaricides; however, excessive reliance on these pesticides is unsustainable due to the widespread resistance in tick populations, as well as an increasing public concern about residues found in animal products, environmental pollution, and the high costs of developing and registering these products (Graf et al., 2004; Ghosh et al., 2007; de la Fuente et al., 2008; Rodriguez-Vivas et al., 2011; Parizi et al., 2012; Merino et al., 2013). Thus, a better understanding of tick biology may greatly improve both current and novel control strategies.

Embryogenesis is one of the most important aspects of *R. microplus* tick life-cycle (Campos et al., 2006; Moraes et al., 2007; Santos et al., 2013). The study of molecules involved in metabolic pathways during embryogenesis could reveal regulatory networks that govern metabolism during this critical stage (Martins et al., 2015). Insulin signaling pathway (ISP) and its components, phosphatidylinositol 3-OH kinase (PI3K), protein kinase B (PKB or AKT) and glycogen synthase kinase 3 (GSK-3), play important roles in metabolic control (Supplementary Figure S1) (de Abreu et al., 2009, 2013; Fabres et al., 2010). In previous studies, we demonstrated that *R. microplus* embryonic cell line, BME26, harbors an insulin-responsive machinery, and that AKT/GSK3 axis integrates glycogen metabolism and cell survival (de Abreu et al., 2009, 2013). We also observed that GSK-3, a key enzyme in glycogen metabolism, is required for cell viability, oviposition, and larval hatching in the cattle tick (Fabres et al., 2010; de Abreu et al., 2013) (Supplementary Figure S1). In order to improve the current knowledge of tick physiology, new targets must be identified and studied.

Target of rapamycin (TOR) signaling pathway (TSP) is regulated by ISP in several animal species (Hay and Sonenberg, 2004; Jia et al., 2004; Wullschleger et al., 2006; Hassan et al., 2013; Hatem et al., 2015; Yoon, 2017). This pathway acts controlling energy metabolism, and monitoring the nutritional status of eukaryotic cells. TSP plays a role in protein synthesis, transcription, cell growth, proliferation, metabolism, aging, and autophagy, from yeasts to mammals (Wullschleger et al., 2006; Guertin and Sabatini, 2007; Jung et al., 2010; Katewa and Kapahi, 2011; Kim and Guan, 2011; Beauchamp and Platanias, 2013). However, the role of TOR target in tick embryogenesis is still unknown.

In the present study, we characterized TSP during *R. microplus* embryogenesis, studying the effects of chemical inhibition on regulation and cell viability *in vitro*. Additionally, to evaluate the dynamics of this pathway *in vivo*, we analyzed the relative transcription of other members of the TOR pathway, S6 kinase (S6K) and eukaryotic translation initiation factor 4E-binding protein 1 (4E-BP1) during embryogenesis. We also describe the effects of TOR gene silencing in partially fed females, particularly in ovarian growth, egg development, and larval hatching. Altogether, our study shows that TSP is important for tick reproductive physiology, that TOR acts as a regulatory target during *Rhipicephalus microplus* embryogenesis and can be considered an important target for tick control.

## Materials and Methods

### Ethics Statement

Animals used in the experiments were housed at Faculdade de Veterinária, Universidade Federal do Rio Grande do Sul (UFRGS) facilities. This research was conducted according to the ethics and methodological guidance, in agreement with the International and National Directives and Norms for Animal Experimentation Ethics Committee of Universidade Federal do Rio Grande do Sul (process number 14403).

### Chemicals

Insulin, TOR inhibitor rapamycin, GSK3 inhibitor alsterpaullone {9-Nitro-7, 12-dihydroindolo [3,2-d][1]benzazepin-6(5H)- one}, MTT [3-(4,5-Dimethyl-2-thiazolyl)-2,5-diphenyl-2H-tetrazolium bromide], and Leibovitz’s 15 culture medium were purchased from Sigma-Aldrich (St. Louis, MO, United States). AKT inhibitor 10-DEBC {10-[4′-(N,N-Diethylamino)butyl]-2-chlorophenoxazinehydrochloride} was purchased from Tocris Bioscience (Ellisville, MO, United States). Fetal bovine serum (FBS) was obtained from Nutricell Nutrientes Celulares (Campinas, SP, Brazil). Other reagents and chemicals used were of analytical grade and locally purchased.

### BME26 Cell Line and *Rhipicephalus microplus* Females and Eggs

BME26 cell line is derived from *R. microplus* embryos at different stages of embryogenesis, first isolated in the 1980s and described by Esteves et al. (2008). Cell cultures were maintained as previously described (Esteves et al., 2008; de Abreu et al., 2013). Leibovitz L15 culture medium (Sigma-Aldrich, United States) was diluted in sterile ultra-pure water (3:1) and supplemented with inactivated fetal bovine serum (20%, Nutricell, Brazil), tryptose phosphate broth (10% Sigma- Aldrich, United States), 100 mL^–1^ penicillin units (Gibco, United States) and 100 μg mL^–1^ streptomycin (Gibco, United States). This fresh complete medium was used for cell culture.

Cells adhered to 25 cm^2^ flasks were resuspended in 5 mL of the medium described above using a 22 gauge needle (0.7 × 25 mm) with the folded tip mounted in a 5 mL plastic syringe. Culture cell density was determined using Neubauer’s chamber (hemocytometer) and cell viability was determined using the trypan blue labeled cell exclusion method (0.4%, Sigma-Aldrich, United States). Aliquots of 1 × 10^7^ cells were transferred to new flasks filled with 5 mL fresh complete medium. The flasks were incubated at 34°C for 2 weeks and the medium replaced weekly to promote cell proliferation. Cells maintained in these flasks, with approximately 4 × 10^6^ cells/mL, were used in later experiments in order to maintain homogenous characteristics between the cells used. Each of these flasks are considered independent biological samples.

*Rhipicephalus microplus* ticks (Porto Alegre strain) were collected from bovines housed in individual tick-proof pens on slatted floors. These ticks are free of pathogens such as *Babesia* spp. and *Anaplasma* spp. (Reck et al., 2009). Partially engorged tick females (20–60 mg each) were manually removed from cattle and used for RNA interference (RNAi) experiments as described below. Two independent experiments were performed, with approximately 20 individuals per treatment for each independent experiment. Fully engorged adult female ticks were collected in Petri dishes and incubated at 28°C and 80% relative humidity until completing oviposition. After oviposition, eggs were separated according to age and maintained under the same conditions. Eggs were collected on different days during the embryogenesis for RNA extraction, determination of vitellin content, or were observed until hatching.

### RNA Extraction and cDNA Synthesis

Total RNA was extracted from BME26 cells (5 × 10^5^ cells in each experimental condition in three biological replicates), from ovaries of partially engorged females in RNAi experiment (three ovaries in each experimental condition in two biological replicates), and from eggs on different days of embryogenesis (50 mg for each day in three biological replicates), using TRIzol^®^ reagent (Invitrogen, Waltham, CA, United States) following the manufacturer’s recommendations. RNA concentration was determined by spectrophotometry at 260 and 280 nm (Picodrop Ltd, United States). Extracted RNA (2 μg) was resuspended in diethylpyrocarbonate (DEPC)-treated water and treated with DNase I (Invitrogen, Waltham, CA, United States). Reverse transcription reaction was performed using High-Capacity cDNA Reverse Transcription Kit (Applied Biosystems, Waltham, CA, United States) following the manufacturer’s protocol, and the resulting cDNA samples were stored at −20°C for analyses of relative transcription.

### Cloning of RmTOR, RmS6K and Rm4E-BP1 ORFs, and Phylogenetic Analysis

RT-PCR was performed using specific primers to clone three open reading frames (ORF): RmTOR, RmS6K, and Rm4E-BP1. The primers (Supplementary Table S1) were designed based on *R. microplus* transcriptome database (RmINCT-EM) created by our research group using Illumina sequencing (unpublished). BME26 cDNA was used as a template. The PCR products were separated by electrophoresis on 1.5% agarose gel and each fragment was excised and purified using GFX^TM^ PCR and Gel Band Purification Kit (GE Healthcare, Chicago, IL, United States). The amplified fragments were ligated into pGEM-T easy vector (Promega) according to the manufacturer’s instructions. Plasmids were transformed into *Escherichia coli* TOP10 cells by electroporation. Recombinant plasmid DNA was extracted using PlasmidPrep Mini Spin Kit (GE Healthcare) and DNA sequencing was performed using T7 and SP6 vector-specific primers (Supplementary Table S1) in 3500 Genetic Analyzer (Applied Biosystems, Hitachi, United States). A total of 4 sequencing efforts were performed for each target. DNA sequence alignment, amino acid translations, and amino acid sequence alignments were done using BioEdit version 7.2.6 software (Hall, 1999). The sequences are listed under GenBank accession numbers: MK598842 (RmTOR), MK598841 (RmS6K), and MK598840 (Rm4E-BP1). Phylogenetic analyses were performed in MEGA software, version 5.05, using coding mRNA sequences (Tamura et al., 2011) and the neighbor-joining method. Bootstrap support was assessed using 1,000 replicates. The sequences included in the phylogenetic analyses are listed under GenBank accession numbers: BAM28764.1 (*Haemaphysalis longicornis);* EFX81736.1 (*Daphnia pulex*); KRT79235.1 (*Oryctes borbonicus*); KZS19304.1 (*Daphnia magna);* XP_002404524.1 (*Ixodes scapularis);* XP_003740398.1 (*Galendromus occidentalis);* XP_008468645.1 (*Diaphorina citri*); XP_013779553.1 (*Limulus Polyphemus);* XP_015792963.1 (*Tetranychus urticae);* XP_015908828.1 (*Parasteatoda tepidariorum);* XP_018027170.1 (*Hyalella Azteca);* NP_477295.1 (*Drosophila melanogaster*); XP_001846418.1 (*Culex quinquefasciatus);* XP_006569762.1 (*Apis mellifera);* and XP_317732.2 (*Anopheles gambiae)*.

### BME26 Cell Treatment With Exogenous Insulin

Cells were treated with exogenous insulin as previously described (de Abreu et al., 2009). BME26 cells were resuspended from flasks in which cell proliferation was promoted. Thereafter, cell density of the culture was determined using Neubauer’s chamber (hemocytometer) and cell viability was determined using the trypan blue-labeled cell exclusion method (0.4%, Sigma-Aldrich, United States). A total of 5 × 10^5^ cells were plated into wells (16.25 mm diameter and 1.93 cm^2^ growth area) of 24 well plates and the final volume of 500 μL was completed with fresh complete medium. Cell plates were incubated at 34°C for a period of 24 h to allow cell adhesion. After this time, complete medium was replaced with medium without fetal bovine serum or with fresh complete medium for 24 h. Subsequently, some cells were exposed to insulin (final concentration 1 μM, Sigma-Aldrich, United States). The plate was incubated at 34°C for 30 min. Finally, all cells were lysed for RNA extraction (described above). All treatments were performed in three independent biological samples and three technical replicates.

### Chemical Inhibition of TOR, AKT and GSK3

BME26 cells were seeded in 24-well plates (5 × 10^5^ cells/well) in 500 μL of complete medium and allowed to settle. After 24 h at 34°C, medium culture was replaced and chemical inhibitors or vehicle control (0.073% DMSO) were added as indicated. BME26 cells were treated with one of the following inhibitors: 0.03 to 2 μM of rapamycin (TOR inhibitor); 12 μM of 10-DEBC (AKT inhibitor, non-lethal concentration); or 40, 400, and 4000 nM of alsterpaullone (GSK inhibitor, non-lethal concentration). After 24 h of incubation at 34°C, cells were processed for total RNA extraction and analysis of S6K and 4E-BP1 relative transcription. Alternatively, cells treated with rapamycin were incubated for 48 h and analyzed for cell viability and membrane integrity as described below. All treatments were performed in three independent biological samples and three technical replicates.

### Cell Viability and Membrane Integrity Assays

After 48 h of TOR chemical inhibition, BME26 cells were subjected to MTT assay (viability assay), following de Abreu et al. (2013). In each well, 50 μL of MTT (12 mM prepared in L15 medium without fetal bovine serum) was added. The plate was incubated at 34°C for 2 h and protected from light. Subsequently, the solution was completely discarded and 1 mL of isopropyl alcohol (0.15% HCl in isopropyl alcohol) was added to each well to dissolve the formazan crystals. The mixture was transferred to 1.5 mL tubes, centrifuged at 6000 × *g* for 15 min and the supernatant was collected in new tubes to measure the absorbance at 570 nm using quartz cuvettes on a UVmini-1240 UV-VIS spectrophotometer (Shimadzu, Japan). The absorbance values of the control treatment (cells treated with 0.073% DMSO) were used for normalization (100% viability). All samples were analyzed in three independent experiments performed in technical triplicates. Additionally, cell viability was determined by trypan blue (0.4%, Sigma-Aldrich, United States) exclusion technique using a Neubauer hemocytometer. The experimental procedure and calculations were performed according to standard methodology (Louis and Siegel, 2011). All samples were analyzed in three independent experiments performed in three technical triplicates.

Membrane integrity in rapamycin-treated BME26 cells was analyzed following de Abreu et al. (2013). Cells were distributed over glass coverslips placed at the bottom of 24-well plate (2 × 10^5^ cells/well). Cells were treated with rapamycin as described above and incubated for 48 h, after which culture medium was gently removed and replaced with PBS. Hoechst 33342 (0.08 mM) was added and incubated for 5 min, followed by addition of propidium iodide (14.8 μM) and further 5-min incubation, always at room temperature and protected from light. The solution was discarded, and coverslips were washed with PBS and mounted over glass slides containing 5 μL of glycerol. BME26 cells were observed under a confocal fluorescence microscope (LSM 780-NLO Zeiss Axio Observer Z.1, Carl Zeiss AG, Germany) using 405 and 488 nm lasers. Images were obtained at 400x magnification.

### Double-Stranded RNA (dsRNA) Synthesis, and Delivery Into BME26 Cells or Female Ticks

A dsRNA based on *R. microplus* TOR coding sequence was designed (dsTOR). Suitable regions of the gene and RNA sequence were screened using BLAST to determine specificity. dsRNA specificity and potential off-targets were estimated from similar genes in other species with the dsCheck program (Naito et al., 2005). *In vitro* dsRNA synthesis was performed using BME26 cDNA as a template and oligonucleotide primers containing T7 promoter sequences (Supplementary Table S2). The amplicon was analyzed by electrophoresis on 1.5% agarose gel, purified using GFX^TM^ PCR and Gel Band Purification Kit (GE Healthcare), and quantified by spectrophotometry at 260 nm (Picodrop Ltd, United States). dsRNA was transcribed *in vitro* from 1 μg of purified cDNA, using the T7 RiboMAX^TM^ Express RNAi System (Promega), and purified according to manufacturer’s instructions. Final yield was estimated by absorbance measurement at 260 nm and quality confirmed by 1.5% agarose gel electrophoresis. The final size of dsRNA was 519 bp. dsRNA synthesis for RmAKT and RmGSK-3β was performed as described previously in Fabres et al. (2010) and de Abreu et al. (2013), respectively. An unrelated 600 bp-long dsRNA of green fluorescent protein (dsGFP) (Supplementary Table S2) was kindly provided by Dr. Albert Mulenga (Texas A&M University, United States).

dsRNA delivery into BME26 cells was performed following de Abreu et al. (2013). A total of 5 × 10^5^ cells were plated into wells (16.25 mm diameter and 1.93 cm^2^ growth area) of 24-well plates, and the final volume of 500 μL was completed with fresh complete medium. Cell plates were incubated at 34°C for a period of 24 h to allow cell adhesion. Subsequently, the culture medium was replaced with 200 μL of fresh medium containing 4 μg of dsRNA and the plate was gently mixed. After 24 h of incubation at 34°C, cells were collected and processed for RNA extraction. RNA from cells treated with dsGFP was used as a control group. All treatments were performed in three independent biological samples and three technical replicates.

dsRNA injection into female ticks was performed according to Fabres et al. (2010). Females with a weight between 20 and 60 mg were manually removed from the cattle and injected with 4 μg of dsRNA in a maximum volume of 2 μL in the lower quadrant of the ventral surface using a Hamilton syringe. Two control groups were injected: one with unrelated dsRNA (4 μg of dsGFP in a maximum volume of 2 μL) and the other with buffer (2 μL of 10 mM PBS, pH 7.4). A third group was injected with TOR dsRNA (4 μg of dsTOR in a maximum volume of 2 μL). The females were fixed on expanded polystyrene plates with double-sided adhesive tape for artificial feeding by capillaries (Gonsioroski et al., 2012). Capillaries were filled with non-infested bovine blood that was collected in heparinized tubes and were replaced every 3 h. The females were maintained with this feeding for 28 h. For confirmation of silencing, ovaries from three females for each independent experiment were collected and processed for RNA extraction after 48 h of dsRNA or PBS injection. Two independent experiments were performed, with approximately 20 individuals per treatment for each independent experiment.

### Analysis of Relative Transcription by qPCR

qPCR reactions were carried out on Applied Biosystems StepOne^TM^ platform, in a total volume of 15 μL, with 100 ng of cDNA, 250 nM of primers (final concentration) and Power Sybr Green Mix (Thermo Fisher, United States), following the manufacturer’s recommendations. Specific primers are described in Supplementary Table S3. The cycling parameters for analyzing RmTOR, RmS6K and Rm4E-BP1 relative transcription were: 10 min at 95°C, followed by 40 cycles of denaturation at 95°C for 15 s and annealing at 60°C for 1 min. For the melt curve stage, the cycling parameters were: 95°C for 15 s, 1 min at 60°C, followed by 35 cycles, with a temperature increase of 0.3°C in each cycle until reaching a final temperature of 95°C. Other cycling parameters for analyzing RmAKT and RmGSK-3β relative transcription were selected according to de Abreu et al. (2013) and Martins et al. (2015). Relative transcription was determined using the Ct values from each run on Relative transcription Software Tool—REST (Pfaffl, 2001). Elongation factor-1A (ELF-1A) was used as a reference gene (Nijhof et al., 2009). These and other features are detailed in Supplementary File S1.

Analysis of relative transcription by qPCR was used in five different experiments: (i) to evaluate relative transcription of RmTOR and RmAKT in BME26 cells treated with exogenous insulin; (ii) to evaluate relative transcription of RmS6K and Rm4E-BP1 after treatment with chemical inhibitors; (iii) to evaluate relative transcription of RmS6K and Rm4E-BP1 after gene silencing following dsRNA treatment; (iv) to evaluate the relative transcription of RmTOR, RmS6K, and Rm4E-BP1 in eggs at different stages of embryogenesis, using cDNA from 1st, 3rd, 5th, 7th, 9th, 12th, 15th, and 18th day of embryogenesis; and (v) to verify gene silencing following dsRNA treatment. All samples were analyzed in triplicates, in three independent experiments, except for dsRNA injection into tick females, which was performed in two independent experiments.

### Determination of Ovarian Growth and Biological Parameters in dsRNA-Treated Females

After 48 h of dsRNA injection, ticks were dissected and 2 ovaries were collected for determination of ovarian growth, using the ovarian growth phase (OGP) system (Seixas et al., 2008). The ovaries were examined in a stereoscope and pictures were analyzed using the software ImageJ (Abràmoff et al., 2004). The proportion of mature oocytes was analyzed by measuring the area occupied by mature and immature oocytes in each image. Additionally, females treated with dsRNA or PBS injection, as well as eggs were collected for examination in a stereoscope and pictures were analyzed.

The biological parameters analyzed were: nutritional efficacy index (final weight of engorged tick/initial weight of engorged tick), eggs production index (EPI) [(weight of eggs/final weight of engorged tick) × 100)], weight of eggs 10 days after oviposition, weight of larvae 40 days after oviposition, and hatching rate (%) (Bennett, 1974; Fabres et al., 2010; Gonsioroski et al., 2012). Two independent experiments were performed, with approximately 20 individuals per treatment for each independent experiment.

### Determination of Vitellin (Vt) Content in Eggs by SDS-PAGE and Dot Blot

Eggs from female ticks treated with dsRNA or PBS injection were collected 9 days after oviposition (10 eggs from each group) and homogenized in 10 mM Tris-HCl buffer, pH 7.4, containing a protease inhibitor cocktail (4EBSF, aprotinin, bestatin hydrochloride, E-64, EDTA, leupeptin hemisulfate salt), pepstatin A (1 mM), and Triton X-100 10%. The samples were kept on ice and immediately used for determination of vitellin content by sodium dodecyl sulfate polyacrylamide gel electrophoresis (SDS-PAGE) and dot blot.

SDS-PAGE was performed under denaturing conditions following the method of Laemmli (1970), followed of silver staining. For dot blot, each sample was spotted onto nitrocellulose membrane (5 μL per spot) and allowed to dry. The membranes were processed as follows: 1 h at room temperature with blocking solution (5% non-fat dry milk/PBS); overnight at 4°C with anti-Vt rabbit serum (1:1000 in blocking solution) (Canal et al., 1995), or blocking solution only, as control; three times 5-min washes with PBS; 1 h at room temperature with secondary antibody (anti-IgG alkaline phosphatase conjugate 1:5000 in blocking solution). Alkaline phosphatase detection was performed with nitro blue tetrazolium (NBT) and 5-bromo4- chloro-3- indolyl phosphate (BCIP) (Promega, United States) in 100 mM Tris pH 9.5 containing 5 mM MgCl_2_ and 100 mM NaCl.

### Statistical Analyses

Unpaired *t*-tests were used for relative transcription analyses, in the insulin-stimulation experiment, chemical inhibition, and gene silencing experiments. ANOVA followed by Dunnett’s test was used in cell viability and cell counting experiments, for analysis of relative transcription during embryogenesis, and for assessment of biological parameters. All statistical analyses were performed using Graph Pad Prism version 6.01 Software (Graph Pad Software, Inc.).

## Results

### TSP Components Are Conserved in *R. microplus*

In this study, we attempted to clone the ORFs of three members of the TSP: RmTOR, RmS6K, and Rm4E-BP1. We were able to sequence a partial RmTOR ORF of 1378 bp (GenBank accession MK598842), which encodes a 458-amino acid polypeptide. Multiple alignment of the deduced amino acid sequence for RmTOR showed 95% identity with the orthologous sequence from the tick *Haemaphysalis longicornis*. We also sequenced a partial RmS6K ORF of 425 bp (GenBank accession MK598841). Multiple alignment of the transcript encoding a S6K protein showed a highly conserved catalytic domain. The identity was 78% with the putative sequence from the tick *Ixodes scapularis*.

The complete sequence of a transcript encoding 4E-BP1 protein was obtained, corresponding to an ORF of 381 nucleotides (GenBank accession MK598840). The encoded protein has 126 amino acids and a predicted molecular weight of 13.3 kDa. Multiple alignment of the deduced amino acid sequence to Rm4E-BP1 identified highly conserved motifs and a high identity level with the orthologous sequences from *H. longicornis* and *I. scapularis* ticks (84 and 74%, respectively).

Phylogenetic analysis was performed using the complete amino acid sequence obtained for Rm4E-BP1 (Supplementary Figure S2). Sequences from 16 species of arthropods were used in the construction of a neighbor-joining tree. The analysis indicates divergences between tick species and other groups of arthropods; for example, sequences from *R. microplus* and *H. longicornis* formed a single clade, while *I. scapularis* sequences were assigned to a distinct clade. Distant clades correspond to insects, such as bees, flies and mosquitoes. Taken together, these results demonstrate that RmTOR, RmS6K, and Rm4E-BP1 are conserved in *R. microplus* tick.

### Exogenous Insulin Can Stimulate RmTOR Relative Transcription

**FIGURE 1:**
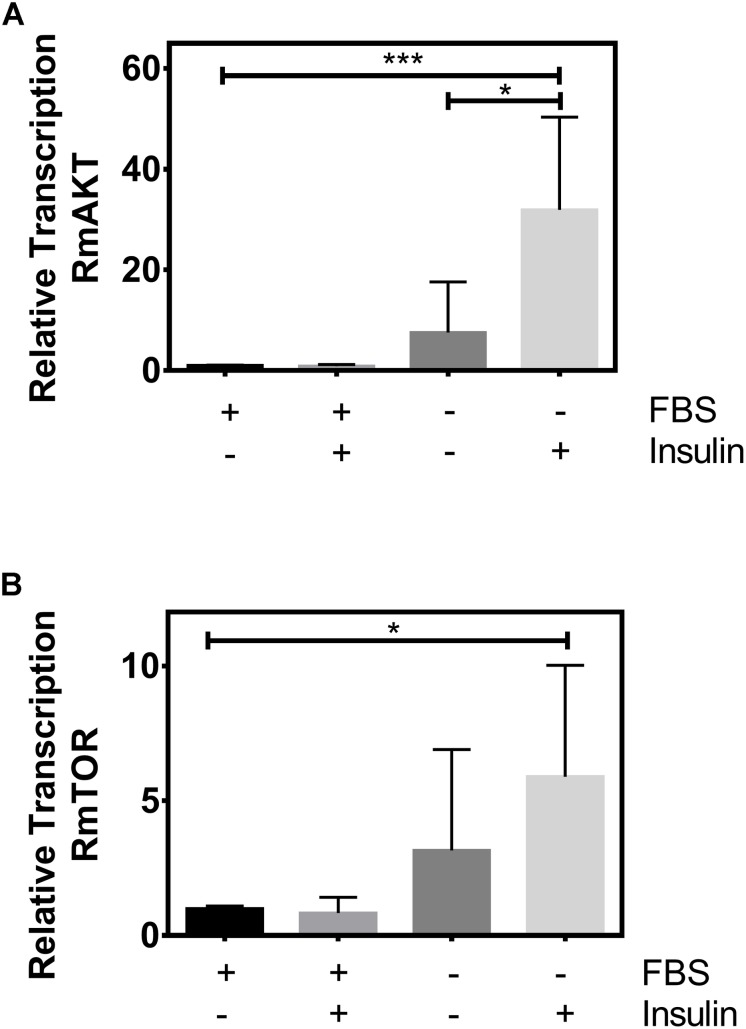
Exogenous insulin stimulates RmAKT and RmTOR relative transcription. BME26 cells were cultured in the absence or presence of fetal bovine serum (FBS), then incubated with or without exogenous insulin (1 μM). Relative transcription of **(A)** RmAKT and **(B)** RmTOR were evaluated using the Pfaffl (2001) method with ELF-1A as reference gene (Nijhof et al., 2009). The plots represent mean ± SD of three independent experiments (^*^p < 0.05; ^∗∗∗^*p* < 0.0001).

ISP regulates TSP in several animal species (Hay and Sonenberg, 2004; Wullschleger et al., 2006; Hassan et al., 2013; Hatem et al., 2015; Yoon, 2017). To assess whether TSP can be regulated/stimulated by insulin in the cattle tick, BME26 embryonic cells were cultured in the absence or presence of fetal bovine serum (FBS), then incubated with or without exogenous insulin (de Abreu et al., 2009). After exposure to insulin, we evaluated the relative transcription of two targets of ISP and TSP: RmAKT and RmTOR (Figure 1).

We observed a higher relative transcription of both RmAKT and RmTOR when cells were incubated with insulin in the absence of FBS compared to cells incubated without insulin and with FBS (normal condition of the cells) (Figure 1). In addition, a statistical difference in RmAKT relative transcription was observed when cells were incubated with insulin in the absence of FBS compared to cells treated without insulin and without FBS (Figure 1A). However, we did not observe a statistical difference in RmTOR relative transcription when cells were incubated with insulin in the absence of FBS compared to cells incubated without insulin and without FBS (Figure 1B).

These results suggest that there is an insulin signaling that stimulates the relative transcription of targets of ISP and TSP in the cattle tick.

### TOR Inhibition Affects Cell Viability and Membrane Integrity in BME26 Cells

**FIGURE 2:**
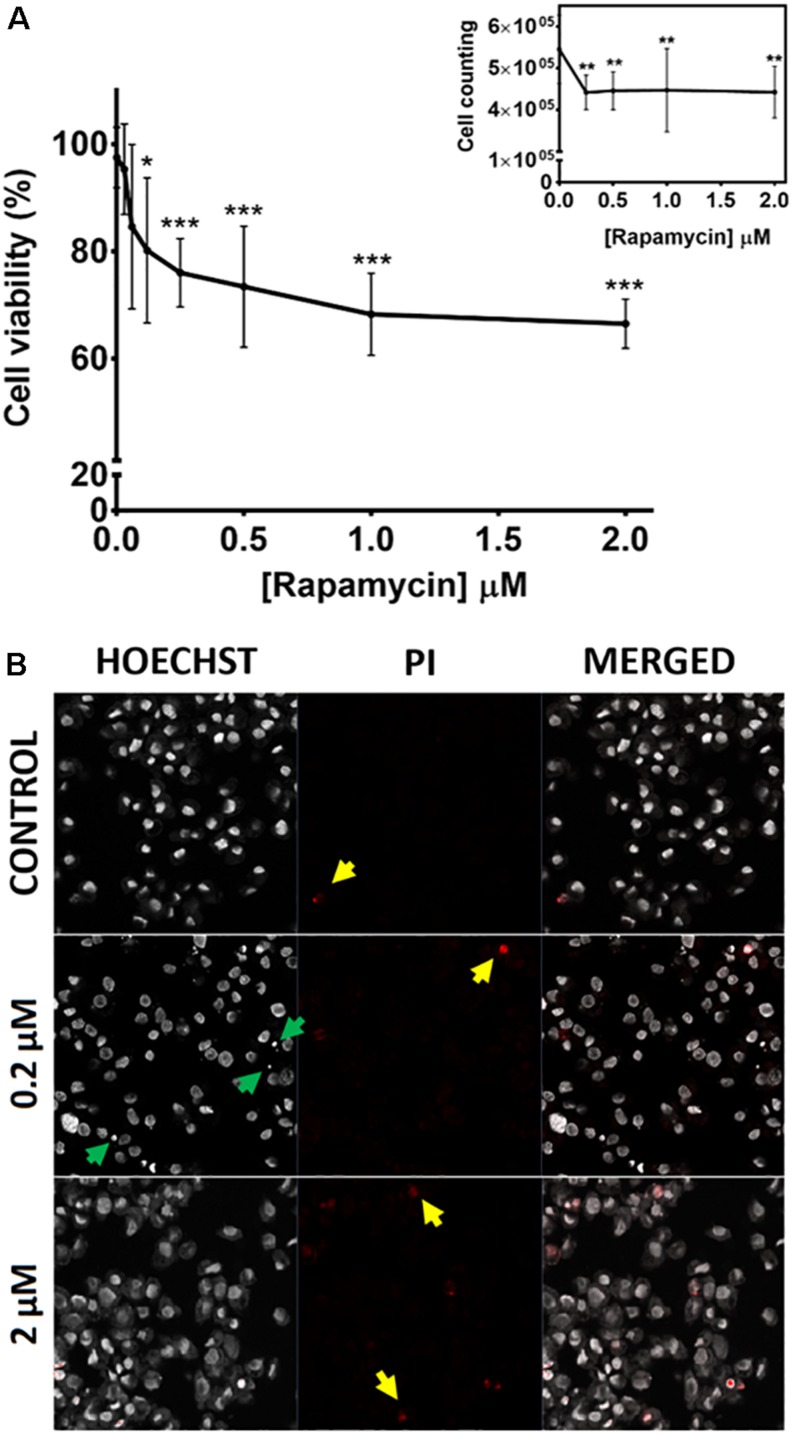
TOR inhibition affects viability and membrane integrity in BME26 cells. BME26 cells were treated with rapamycin (0.03 to 2 μM) for 48 h. Cells treated with 0.073% DMSO were used as the control group. **(A)** Cell viability was analyzed by MTT assay and cell counting (insert). Results were normalized to control exposed to vehicle alone (DMSO). Plots present mean ± SD of three independent experiments (^*^p < 0.05; ^∗∗^*p* < 0.005; ^∗∗∗^*p* < 0.0001). **(B)** Membrane integrity of the treated cells was evaluated following de Abreu et al. (2013). Cells were stained with Hoechst 33342 (left column) and propidium iodide (center column and yellow arrows). Events of pyknosis are indicated by green arrows. Images were obtained in a confocal fluorescence microscope at 400x magnification.

Given the fundamental role played by TOR in cell viability in other species, we studied the effects of TOR chemical inhibition in the cattle tick, using tick embryonic BME26 cells. After treatment with different concentrations of rapamycin for 48 h, cell viability was evaluated by MTT assay (Figure 2A) and cell counting (Figure 2A, insert). Rapamycin concentrations above 0.12 μM caused significant reduction in cell viability, reaching 34% decrease at 2 μM, compared to control cells (cells treated with 0.073% DMSO) (Figure 2A); accordingly, we observed a significant reduction in cell density at rapamycin concentrations between 0.25 and 2 μM compared to control cells (Figure 2A insert).

Additionally, membrane integrity was analyzed in BME26 cells upon rapamycin treatment (Figure 2B). Rapamycin concentrations above 0.2 μM caused the cells to stain more intensely with propidium iodide, compared to control cells (red staining highlighted with yellow arrows in Figure 2B). Cells with intact membrane are generally not permeable to propidium iodide, therefore the intense staining indicated membrane damage. BME26 cells treated with rapamycin also exhibited chromatin condensation (pyknosis, indicated by green arrowheads in Figure 2B), a possible indication of cell death by apoptosis. Taken together, these results suggest that TOR is important for cell viability in BME26 cells.

### TOR Regulates S6K and 4E-BP1 in BME26 Cells

**FIGURE 3:**
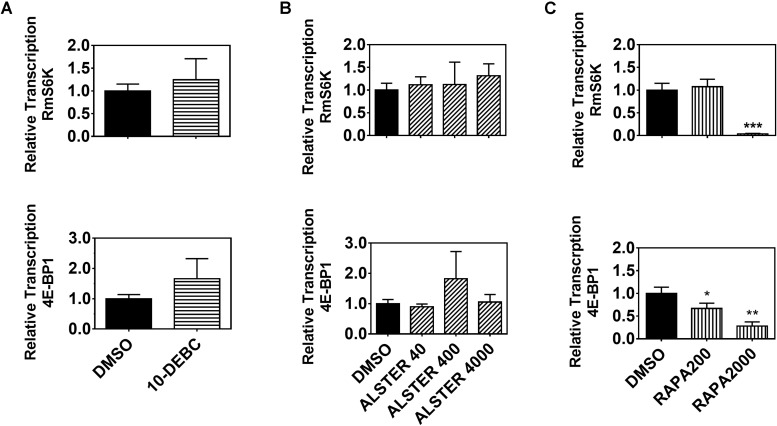
Transcription of downstream ISP/TSP targets requires TOR enzymatic activity. Relative transcription of two downstream targets (S6K and 4E-BP1) was evaluated 24 h after BME26 cells were treated with chemical inhibitors of: **(A)** AKT (10-DEBC 12 μM), **(B)** GSK (alsterpaullone 40, 400 and 4000 nM), or **(C)** TOR (rapamycin 200 and 2000 nM) or vehicle (0.073% DMSO) as control. Relative transcription was calculated according to the Pfaffl (2001) method using ELF-1A as reference gene (Nijhof et al., 2009), and data were analyzed in unpaired t-test. The plots present mean ± SD from three independent experiments (^*^p < 0.05; ^∗∗^*p* < 0.005; ^∗∗∗^*p* < 0.0001).

To further investigate the possible interaction between ISP and TSP components in *R. microplus*, we performed gene silencing of three signaling components (RmAKT, RmGSK-3β and RmTOR) in BME26 cells. First, we confirmed gene silencing by qPCR (Supplementary Figure S3) and evaluated the relative transcription of downstream targets (Supplementary Figure S4). Gene silencing efficiency was 75% for RmAKT, 45% for RmGSK-3β, and 80% for RmTOR (Supplementary Figure S3). No difference was observed in the transcription of any of these targets upon dsRNA treatment (Supplementary Figure S4). However, a contrasting result was observed when using chemical inhibitors of each of the signaling components, namely 10-DEBC (AKT inhibitor), alsterpaullone (GSK inhibitor), and rapamycin (TOR inhibitor). Rapamycin treatment caused reduced transcription levels of RmS6K and Rm4E-BP1 when compared to control treatment (Figure 3C). In contrast, AKT and GSK-3β chemical inhibition (Figures 3A,B) did not cause any detectable difference in the relative transcription of the downstream targets. Taken together, these results lead us to believe that TOR regulates S6K and 4E-BP1 in BME26 cells.

### RmTOR Is Transcribed During Tick Embryogenesis

**FIGURE 4:**
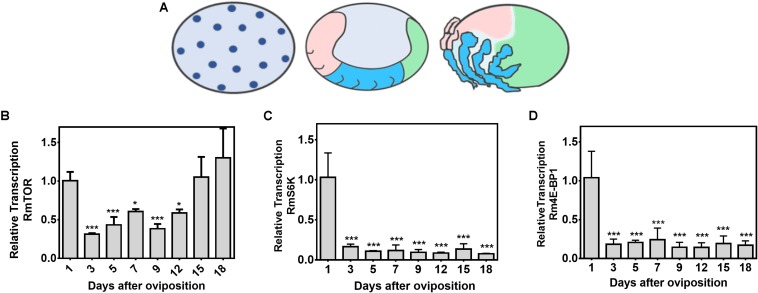
RmTOR, but not RmS6K and Rm4E-BP1, is transcribed at different stages during tick embryogenesis. The different stages of *R. microplus* embryo development (according to Santos et al., 2013) are depicted in **(A)** (initial stage; middle stage; and final stage). Relative transcription of RmTOR **(B)**, RmS6K **(C)**, and Rm4E-BP1 **(D)** was determined by qPCR using cDNA from eggs collected on different days of embryogenesis. Relative transcription was calculated according to the Pfaffl (2001) method using ELF-1A as reference gene (Nijhof et al., 2009), and data were analyzed by one-way ANOVA, followed by Dunnett’s test, where all samples were compared with 1-day-old eggs. Plots present mean ± SD from three independent experiments (^*^p < 0.05; ^∗∗∗^*p* < 0.0001 compared with first day after oviposition).

With the main goal of investigating the role of TSP in *R. microplus* embryogenesis, we evaluated the transcriptional profile of RmTOR, RmS6K, and Rm4E-BP1 during embryo development (Figure 4). Developmental stages are depicted in Figure 4A: the initial stage represents the 1st to 3rd day after oviposition (DAO), characterized by cleavages starting inside the yolk and further cell divisions; the middle stage corresponds to the end of 6th to 7th DAO, characterized by the beginning of germ band extension and generation of abdominal segments; the final stage comprises the 15th to 18th DAO, characterized by a nearly complete dorsal closure. The highest level of RmTOR relative transcription was observed in the first day, decreasing to about half the initial levels in the subsequent days, then surging again in the final developmental stage, between days 15 and 18 (Figure 4B). On the other hand, RmS6K and Rm4E-BP1 transcriptional levels, which were also highest on the first day after oviposition, markedly decreased during all the remaining of the embryogenesis period (Figures 4C,D). The data might indicate a role for TOR during tick development.

### RmTOR Gene Silencing in Female Ticks Affects Ovarian Development, Vitellin Content in Eggs, and Egg Hatching

**FIGURE 5:**
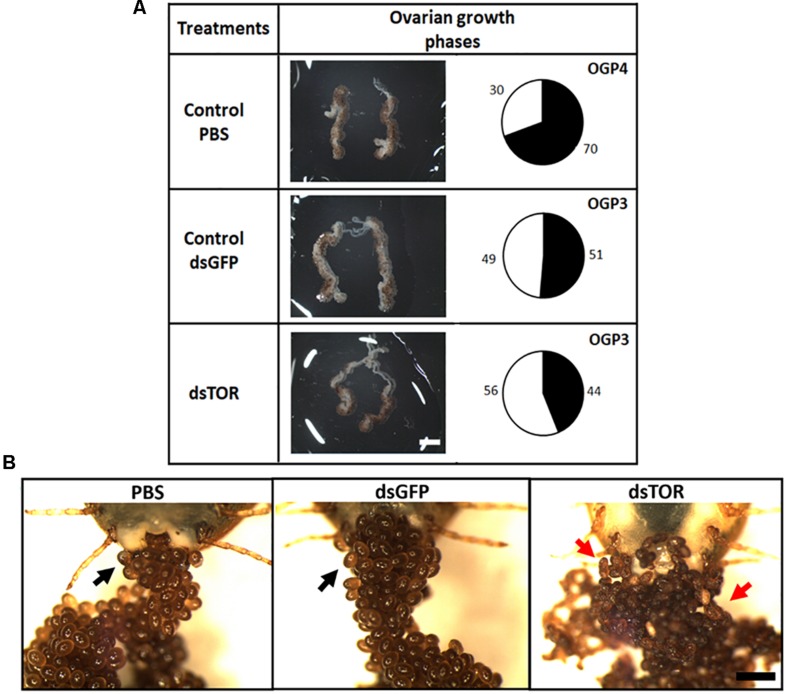
TOR gene silencing delays ovarian development and affects oviposition in *R. microplus*. **(A)** Females were dissected 48 h after dsRNA treatment for optical assessment of ovarian development. Ovaries were examined in a stereoscope. The relative proportion of mature to immature oocytes was analyzed by area measurements, as described in Section “Materials and Methods.” Proportion of area occupied by developed oocytes is indicated in pie charts (black). Scale bar: 2 mm. **(B)** Representative images show the effect on oviposition of dsRNA injection (dsTOR, dsGFP or PBS control) in partially engorged female ticks. Eggs were examined under stereoscope 10 days after the start of oviposition. Normal eggs are indicated by black arrows and atypical eggs are indicated by red arrows. Scale bar: 1 mm.

RmTOR was silenced by RNAi in partially engorged female ticks in order to study its role during *R. microplus* embryogenesis. We observed lower levels of TOR transcripts in ovaries from ticks treated with RmTOR-dsRNA compared to dsGFP-injected control (gene silencing efficiency was 77%) (Supplementary Figure S5). After 48 h of injection, ovaries dissected from females treated with dsTOR showed a slightly delayed development compared with controls (Figure 5A). According to the ovarian growth phase (OGP) system proposed by Seixas et al. (2008), dsTOR-injected females had ovaries classified as stage 3, while ovaries from the PBS control group were classified as stage 4. Also, ovaries from dsTOR group showed a reduced proportion of mature follicles (Figure 5A, black area in pie charts).

**FIGURE 6:**
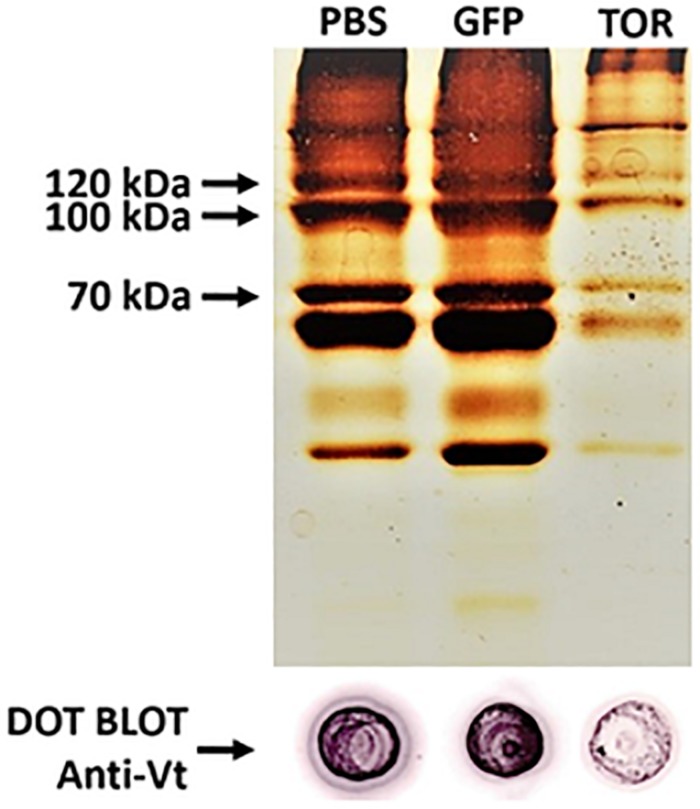
TOR gene silencing decreased vitellin (Vt) content in eggs. Eggs from female ticks treated with dsRNA injection (PBS, dsGFP and dsTOR) were collected 9 days after oviposition (middle stage). The samples were used for determination of vitellin content by SDS-PAGE **(top)** and dot blot **(bottom)**. Arrows on the **(top)** panel indicate the approximate molecular weight of the three major VT subunits (120, 105, and 70 kDa).

Eggs laid by females treated with dsTOR showed an atypical external appearance when observed on day 10 after the start of oviposition, in contrast to the typical morphology observed in eggs from the control groups (PBS and dsGFP) (Figure 5B). Recognizing that vitellogenesis is a fundamental process in tick development which can be regulated by TSP via S6K (Umemiya-Shirafuji et al., 2012), we investigated vitellin content in eggs from females subjected to gene silencing (Figure 6). Eggs from female ticks treated with dsRNA or PBS injection were collected 9 days after oviposition, when important morphological and biochemical events occur, including: the final development of the germ band, formation of the abdominal segments, early formation of the four pairs of legs. Also at this stage, a great reduction in carbohydrate content takes place, suggesting that gluconeogenic processes are necessary to sustain egg development (Moraes et al., 2007; Logullo et al., 2009; Santos et al., 2013; Martins et al., 2018). SDS-PAGE, followed by densitometric analyses of the three major VT subunits (120, 105, and 70 kDa), and dot blot demonstrated a lower vitellin content in eggs laid by dsTOR-treated females when compared with controls. Accordingly, we also observed a lower total protein content in these eggs.

**FIGURE 7:**
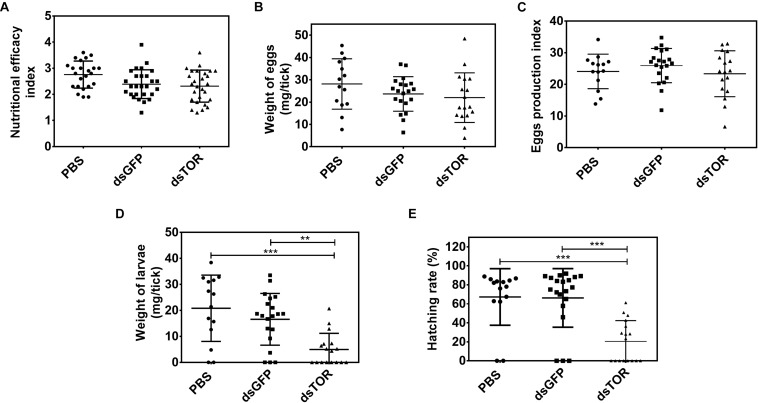
TOR gene silencing affects egg hatching in *R. microplus*. Biological parameters were analyzed after dsRNA treatment (dsTOR, dsGFP, or PBS control) in partially engorged female ticks. **(A)** Nutritional efficacy index; **(B)** weight of eggs (mg/tick); **(C)** egg production index (EPI); **(D)** weight of larvae (mg/tick); and **(E)** hatching rate (%). Plots present mean ± SD from two independent experiments (^∗∗^*p* < 0.005; ^∗∗∗^*p* < 0.0001).

The nutritional efficacy index was not affected by RmTOR gene knockdown (Figure 7A), indicating that dsRNA injection did not affect feeding behavior. Also, egg weight and the egg production index remained unaffected in dsTOR-injected group (Figures 7B,C). In contrast, a significant reduction in the weight of larvae and in hatching rate was observed after TOR gene silencing in comparison with control groups after 40 days (Figures 7D,E). Taken together, these results led us to believe that TOR is important for tick reproduction.

## Discussion

TSP is a central regulatory pathway in energy metabolism, and nutritional status monitoring in eukaryotic cells. TSP is fundamental for protein synthesis, transcription, cell growth, cell survival, proliferation, aging, and autophagy, from yeast to vertebrates (Hawkins et al., 2006; Wullschleger et al., 2006; Guertin and Sabatini, 2007; Jung et al., 2010; Katewa and Kapahi, 2011; Kim and Guan, 2011; Laplante and Sabatini, 2012; Beauchamp and Platanias, 2013; Na et al., 2015; Kennedy et al., 2016). This paper demonstrates that the role of TOR is conserved in ticks and fundamental in the embryogenesis of the cattle tick *R. microplus*.

In order to investigate components of TSP in the cattle tick *R. microplus*, we sequenced and analyzed either partial or complete nucleotide sequences coding for RmTOR, RmS6K and Rm4E-BP1.

TOR is an atypical serine/threonine kinase of the phosphatidylinositol kinase-related kinase (PIKK) family, that comprises two conserved complexes, named TOR complex 1 (TORC1) and TOR complex 2 (TORC2) (Hay and Sonenberg, 2004; Wullschleger et al., 2006; Soulard et al., 2009; Loewith, 2011; Laplante and Sabatini, 2012; Sabatini, 2017). TORC1 acts as an integrator of extracellular and intracellular signals, such as the nutrient availability, growth factors, and stress energy levels (Hay and Sonenberg, 2004; Reiling and Sabatini, 2006; Wullschleger et al., 2006; Zoncu et al., 2011). These signals can be cooperative or antagonists, enabling the cell to adjust and perform appropriate responses for each condition (Howell and Manning, 2011). This TOR complex stimulates S6 kinase (S6K) activity, as well as phosphorylating factor 4E-Binding Protein 1 (4E-BP1) that leads to the release of eukaryotic initiation factor 4E (eIF4E), thus initiating translation of specific mRNAs (Engelman et al., 2006; Shaw and Cantley, 2006; Ma and Blenis, 2009). A domain associated with the complex regulator known as Raptor (regulatory-associated protein of mTOR) defines mTORC1, and is inhibited by rapamycin, a property that originated the protein name, ‘Target Of Rapamycin’ (Loewith, 2011).

On the other hand, the less studied TORC2 has been considered to be insensitive to nutrient levels, but responsive to growth factor signaling, possibly by associating with translating ribosomes in response to growth factor receptor-PI3K activation (Oh et al., 2010; Zinzalla et al., 2011), and to function mainly by activating protein kinase B (AKT) via Ser473 phosphorylation (Sarbassov et al., 2005; Masui et al., 2014). It can also phosphorylate other protein kinase A, G, and C families (AGC kinases). TORC2 may also be regulated by amino acids, depending on specific substrates and cellular context. Ribosomes have been reported to be direct activators of mTORC2 in response to insulin (Zinzalla et al., 2011; Takahara and Maeda, 2013).

Target of rapamycin protein is structurally defined by the presence of several conserved domains such as the HEAT repeat, focal adhesion target (FAT), FKBP12/rapamycin binding (FRB), kinase, and FAT C-terminal (FATC) domains, starting from the N-terminus (Yang et al., 2013; Maegawa et al., 2015). In the cattle tick *R. microplus*, the amino acid sequence of RmTOR is composed of three classical domains: FRB domain, a catalytic domain, and FATC domain. In the amino acid sequence of RmS6K, we found a conserved catalytic domain (Hanks and Quinn, 1991; Hunter, 1991). Investigating the identity of RmTOR and RmS6K with orthologous sequences, a high identity with other ticks was observed, proving the conservation of these proteins.

From full-length 4E-BP1 cDNA, the deduced amino acid sequence presented three motifs: RAIP, TOS, and YXXXXLφ, important regulatory sites highly conserved among invertebrate and vertebrate organisms (Mader et al., 1995; Lawrence and Abraham, 1997; Gingras et al., 1999; Schalm and Blenis, 2002; Tee and Proud, 2002). It is worth noting that three isoforms were identified from vertebrates (Pause et al., 1994; Rousseau et al., 1996; Poulin et al., 1998), while only one isoform was identified in arthropods (Gutzeit et al., 1994; Bernal and Kimbrell, 2000; Lasko, 2000; Cormier et al., 2001; Miron et al., 2001; Gu et al., 2011; Kume et al., 2012). In the present study, only one 4E-BP1 isoform was identified from *R. microplus*, and showed high identity with the orthologous sequences from *H. longicornis* and *I. scapularis* ticks.

Recognizing that the TSP is conserved in *R. microplus*, our main goal was to investigate the role of this pathway in *R. microplus* embryogenesis. Therefore, we evaluated the transcriptional profile of RmTOR, RmS6K, and Rm4E-BP1 during embryo development (Figures 4B–D). Transcriptional levels of RmS6K and Rm4E-BP1 were highest on the first day after oviposition, which suggests that these transcripts are of maternal origin. In the tick *H. longicornis*, TOR controls vitellogenesis via activation of S6 kinase (S6K) in pre-ovipositional stage (Umemiya-Shirafuji et al., 2012), and 4E-BP1 is highly relevant for lipid storage during the non-feeding starvation period (Kume et al., 2012). S6K and 4E-BP1 have also been implicated in embryo development in other models. For example, in *Xenopus laevis*, S6K activity is high immediately after fertilization, presumably for altering the translational capacity available for mRNAs lacking a 5′-TOP region (Schwab et al., 1999). Also, in sea urchin embryos, dissociation of 4E-BP1 of its target, the eukaryotic initiation factor 4E (eIF4E), is functionally important for the first mitotic division (Salaün et al., 2004; Salaun et al., 2005). In the mosquito *Aedes aegypti*, RNA-interference-mediated depletion of S6K demonstrated that the TOR pathway is required for mosquito egg development (Hansen et al., 2005), while depletion of 4E-BP1 affects mosquito longevity and is critical in modulating translational events that are dependent on nutritional, developmental and stress conditions (Roy and Raikhel, 2012).

RmTOR transcriptional profile demonstrated a large mRNA deposition by the mother in 1-day-old eggs, followed by lower levels during the middle stage of embryogenesis. Toward the final stage, when dorsal closure is undergoing, TOR transcript levels rise again to levels equivalent to the initial stage (Figure 4B). In mosquitoes, TOR serves as a key regulator to complete vitellogenesis (Hansen et al., 2004, 2005). Previous studies showed that chemical inhibition of TOR with rapamycin or RNAi-mediated gene depletion resulted in significant down-regulation of vitellogenin transcription after amino acid stimulation in fat body culture system *in vitro*, as well as in inhibition of egg development *in vivo* (Hansen et al., 2004, 2005). In *Drosophila*, TSP activation promotes yolk catabolism in embryos (Kuhn et al., 2015). It remains to be determined if these *R. microplus* TSP components function in similar ways.

To further elucidate the role of TOR in *R. microplus* embryogenesis, two strategies were employed in this study: chemical inhibition in tick embryonic cell line (BME26), and RNAi in partially engorged female ticks. It is known that the study of TSP functions in invertebrate and vertebrate organisms has been greatly advanced by exploring the mechanism of action of rapamycin, a specific inhibitor of TOR, which has several clinical applications as immunosuppressive, antifungal, and anticancer (Shor et al., 2009; Thoreen et al., 2009; Wu and Hu, 2010; Zaytseva et al., 2012). BME26 cells treated with concentrations of rapamycin greater than 0.12 μM showed a significant decrease in cell viability and cell counting (Figure 2A). Furthermore, we observed that BME26 cells treated with concentrations greater than 0.2 μM showed a higher intensity of staining with propidium iodide compared with control cells, and exhibited chromatin condensation (Figure 2B). Similar results have been described for T lymphocytes and endothelial cells (Barilli et al., 2008; Wang et al., 2012). It has been shown that rapamycin causes G1 arrest and blocks G1 to S phase transition in fibroblasts and B-CLL cells (Hashemolhosseini et al., 1998; Decker et al., 2003). It can also cause dysregulation of the two effector proteins of the TSP pathway, S6K and 4E-BP1, causing negative impact in cap-dependent initiation of translation (Beretta et al., 1996), in cellular migration (Poon et al., 1996), and activating cell death by apoptosis or necrosis (Shi et al., 1995; Barilli et al., 2008). Our results demonstrate that TOR directly affects cell viability in tick embryonic cells.

To assess whether TSP can be regulated/stimulated by insulin in the cattle tick, BME26 embryonic cells were exposed to insulin, and the relative transcription of two components of ISP and TSP pathways (AKT and TOR) was evaluated (Figure 1). We observed a higher relative transcription of TOR when cells were incubated with insulin in the absence of FBS (Figure 1B), indicating that TOR can be stimulated by exogenous insulin. This interaction between the two pathways has also been demonstrated in other species (Hay and Sonenberg, 2004; Wullschleger et al., 2006; Hassan et al., 2013; Hatem et al., 2015; Yoon, 2017). Also, AKT relative transcription was higher when cells were incubated with insulin in the absence of FBS when compared to control cells (Figure 1A), an even more marked increase than observed for TOR. This can be attributed to the direct regulation of AKT by insulin (de Abreu et al., 2009, 2013). In previous studies, we demonstrated that *R. microplus* embryonic cell line (BME26) displays an insulin-responsive machinery, and that AKT/GSK3 axis integrates glycogen metabolism and cell survival (de Abreu et al., 2009, 2013). Likewise, our results suggest that ISP can stimulate/regulate TSP in the tick *R. microplus*.

Target of rapamycin gene silencing in partially engorged females caused a slight delay in ovary development (Figure 5A), an atypical external appearance in 10-day-old eggs (Figure 5B), lower vitellin and protein content (Figure 6), and decreased hatching rate in comparison with control groups (Figure 7), results that altogether suggest TOR is important for tick embryo development and tick reproduction. Collectively, our data agree with studies developed in other arthropods. For example, in *Drosophila*, using maternal short hairpin RNAs technology (shRNA), it was observed that shRNA-TOR embryos were smaller than control embryos, and showed significant DNA fragmentation post-cellularization (Kuhn et al., 2015). According to a recent report in *Lepeophtheirus salmonis*, RNAi mediated knockdown of TOR pathway genes resulted in inhibition of egg development and maturation (Sandlund et al., 2018). In other models, TSP has been studied for its role in oogenesis or/and vitellogenesis, for example in the red flour beetle *Tribolium castaneum* (Parthasarathy and Palli, 2011), the brown planthopper *Nilaparvata lugens* (Zhai et al., 2015; Lu et al., 2016), in the mosquito *Aedes aegypti* (Hansen et al., 2004, 2005), and in *H. longicornis* tick (Umemiya-Shirafuji et al., 2012). However, this is the first study reporting the importance of TSP during embryogenesis in ticks. It has been demonstrated that, in several insect species, ISP and TSP serve as important sensors of nutritional status and are required for initiation of reproductive events, such as oocyte maturation and vitellogenin synthesis. Previous work by our group demonstrated that vitellin, the main tick yolk protein, is internalized by the oocyte (Seixas et al., 2018) and it is a reservoir of heme for embryo development (Logullo et al., 2002). We show in the present study that TOR silencing decreases vitellin content in eggs 9 days after oviposition (Figure 6), which means that development will likely be affected. Accordingly, we also observed a lower total protein content in eggs laid by dsTOR-treated females when compared with controls; this may be related to the fact that TOR is involved in the processes of ribosomal biogenesis and protein biosynthesis (Oh et al., 2010; Zinzalla et al., 2011).

Finally, with the aim of studying the interconnection between ISP/TSP signaling pathways in *R. microplus*, we investigated the transcription of downstream targets (S6K and 4E-BP1) in BME26 embryonic cell line treated with chemical inhibition or RNAi of upstream regulators (Figure 4 and Supplementary Figure S3). The results show a lower relative transcription for S6K and 4E-BP1 when treated with rapamycin, in comparison with untreated cells, demonstrating a consistent inhibitory effect and suggesting a potentially conserved function of TSP pathway in ticks (Figure 3). Rapamycin treatment has allowed the investigation of TSP roles in diverse processes, for example: regulation of juvenile hormone and expression of vitellogenin in the cockroach *Blattella germanica* (Maestro et al., 2009); deposition of proteins important for embryogenesis in blood-fed *A. aegypti* females (Gulia-Nuss et al., 2011; Pérez-Hedo et al., 2013); and regulation of S6K and 4E-BP proteins in different organisms (Stewart et al., 1996; Montagne et al., 1999; Oldham et al., 2000; Umemiya-Shirafuji et al., 2012). In contrast, we did not observe any difference in the relative transcription of downstream targets in BME26 cells treated with chemical inhibitor of AKT or GSK3, suggesting that, unlike TOR, these proteins are not involved in S6K and 4E-BP1 regulation. When BME26 cells were treated with RNAi for AKT, GSK3, or TOR, the transcription of downstream targets seemed to be unaffected (Supplementary Figure S4), suggesting that the effect of gene silencing was not sufficient to affect protein function, and that regulation occurs mainly at the post-transcriptional level, as classically described in kinase pathways (Woodgett, 2001; Riehle and Brown, 2003; Hay and Sonenberg, 2004; Sarbassov et al., 2005; Albert and Hall, 2015).

The cattle tick *R. microplus* is an obligate hematophagous arthropod that poses a serious threat to dairy and cattle production. Amongst the indirect effects of its diet is the transmission of various pathogens, and the resulting diseases can cause large losses in livestock production, reduce farm incomes, increase costs for consumers, and threaten trade between regions and/or world markets (Tabor et al., 2017). Strategies for the control of diseases transmitted by this arthropod vector have been managed mainly with two general approaches: (i) focusing on interventions, such as vaccines or drug treatments, and (ii) focusing on the development of strategies based on vector knowledge, aimed at reducing transmission by reducing vector density or interfering with the vector’s ability to transmit the disease-causing pathogen (Murray et al., 2012). Our research group focuses on this second strategy, aiming to contribute knowledge to the understanding of *R. microplus* tick biology, studying mainly the aspects related to energy metabolism during embryogenesis. ISP/TSP signaling pathways are the main bridge between nutrient detection system and development of different cellular processes, such as cell viability, growth, and proliferation in arthropods (Grewal, 2009; Benmimoun et al., 2012; Pérez-Hedo et al., 2013).

Here, we demonstrate that the role of TOR is conserved in ticks and fundamental for tick reproductive physiology. Combined with previous work by our group (de Abreu et al., 2013), we show that TOR is a regulatory target in *R. microplus* embryogenesis and can be considered an important target for tick control. Future studies should be able to understand the side effects of chemical inhibition in other cellular processes in the cattle tick.

## Data Availability

The datasets generated for this study can be found in GenBank with the accession numbers MK598842, MK598841, and MK598840.

## Ethics Statement

Animals used in the experiments were housed at Faculdade de Veterinária, Universidade Federal do Rio Grande do Sul (UFRGS) facilities. This research was conducted according to the ethics and methodological guidance, in agreement with the International and National Directives and Norms for Animal Experimentation Ethics Committee of Universidade Federal do Rio Grande do Sul (Process No. 14403).

## Author Contributions

CW, LA, and CL conceived and designed the experiments. CW and TA performed the experiments. CW, RN-d-F, ISV, and CL contributed reagents, materials, and analysis tools. CW, LA, RN-d-F, ISV, and CL drafted the manuscript. CW, LA, RN-d-F, ISV, and CL critically revised the manuscript.

## Conflict of Interest Statement

The authors declare that the research was conducted in the absence of any commercial or financial relationships that could be construed as a potential conflict of interest.
