# Supplementary material for: TOR as a Regulatory Target in Rhipicephalus microplus Embryogenesis
Source: Front Physiol. 2019 Jul 31;10:965. doi: 10.3389/fphys.2019.00965 (PMC6684781; doi:10.3389/fphys.2019.00965)

Supplementary Material

Table S1. Primers used in the cloning and sequencing of TOR, S6K, and 4E-BP1 open reading frames from the tick *Rhipicephalus microplus*.

TARGET	PRIMER SEQUENCE (5'- 3')	FRAGMENT SIZE (bp)
TOR	FWD: GTACCAGCGTTCCTTGAATGTCAAAG<br>REV: CCCTTTCTAGCACCTCTCAACAGGAC	1378
4E-BP1	FWD: TGGGCGCTCGTGGAGAACG<br>REV: CTGGTGGCACTCCGTACTGGAGC	381
S6K	FWD: AGGACCTCTGTCCTCATGACGTCC<br>REV: CTCGAAGTCTTGCTCATGATTTCCAC	425
T7 UNIVERSAL PRIMER	TAATACGACTCACTATAGGG	-
SP6 UNIVERSAL PRIMER	ATTTAGGTGACACTATAG	-

Table S2. Primers used for RNAi-mediated AKT, GSK3-β, and TOR silencing in *Rhipicephalus microplus.*

Target	Primer sequence (5'- 3')	Fragment size (bp)	Reference
AKT	FWD: taatacgactcactatagggTCAGCCTGGACAACTTTGAGTTCCT<br>REV: taatacgactcactatagggATTTCATACATGACCACGCCCAGC	595	(de Abreu et al., 2013)
GSK3-β	FWD: taatacgactcactatagggTTATGCGACGGCTAGAACACT<br>REV: taatacgactcactatagggGCTCTTGCTCTGTGAAGTTGAA	798	(Fabres et al., 2010)
TOR	FWD: taatacgactcactatagggGAGGTGACTGGCATTGAG<br>REV:taatacgactcactatagggGCTTGTGGACGCATCTTC	519	This article
GFP	FWD: taatacgactcactatagggTCACGAACTCCAGCAGGACCATGTGATC<br>REV: taatacgactcactatagggACGTAAACGGCCACAAGTTCAGCGTGTC	600	(Mulenga and Khumthong, 2010)

Table S3. qRT-PCR primers used for quantification of different gene transcripts in *Rhipicephalus microplus*.

Target	Primer sequence (5'- 3')	Fragment size (bp)	Reference
AKT	FWD: GAAGTTGGCGAGTGAGGAGGA<br>REV: CGCGTCGAGATGCTGAACTTGT	86	(de Abreu et al., 2013)
GSK3-β	FWD: CCCACACCCGCTATTTATTG<br>REV: TGTGCAGGAGAGCCAGTTTA	113	(Martins Da Silva et al., 2015)
TOR	FWD: AGGCTCTGAACAAAAAGGC<br>REV: GAACATCCAAAGTCTCATCTG	93	This article
4E-BP1	FWD: CCAGATGTCTCCAAGGCCAG<br>REV: GTTCGTCACCCGATCCTTCA	103	This article
S6K	FWD: TGTGATTGGTGGAGTTTTGG<br>REV: GCGAGTGCTTGGTGTAAC	96	This article
ELF-1A	FWD: CGTCTACAAGATTGGTGGCATT<br>REV: CTCAGTGGTCAGGTTGGCAG	108	(Nijhof et al., 2009)

Fig. S1. Schematic representation of insulin signaling pathway (ISP) and TOR signaling pathway (TSP). The two pathways were demonstrated to be conserved in arthropods and some of its key components are represented. Solid and dashed arrows represent one-step and multi-step events, respectively; rounded arrowhead indicates inhibition; inhibitors for each target are presented in italicized text. Abbreviations: PI3K, phosphatidylinositol 3-OH kinase; AKT, protein kinase B; GSK-3β, glycogen synthase kinase 3 beta; TOR, target of rapamycin; S6K, S6 kinase; 4E-BP1, eukaryotic translation initiation factor 4E – binding protein 1. Previously described functions of these signaling molecules in *R. microplus* are presented in grey boxes. The role of TOR in tick embryogenesis is still unknown.

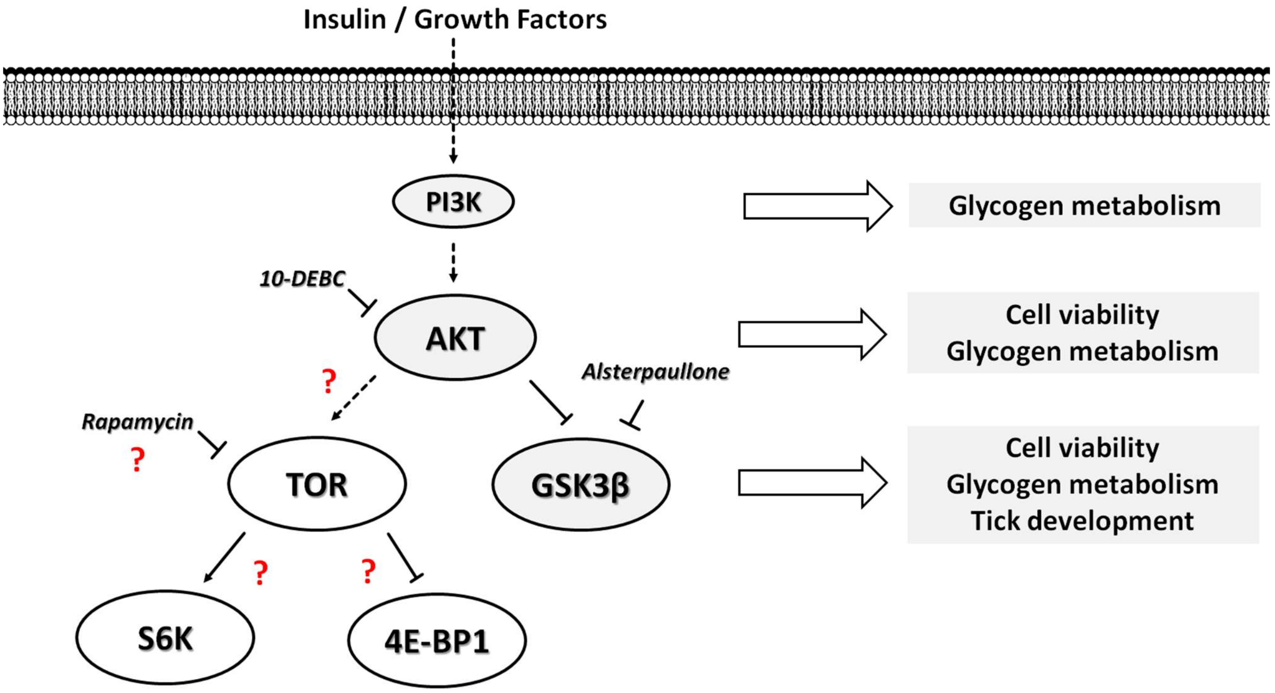

Fig. S2. Phylogenetic tree of *Rhipicephalus microplus* Rm4E-BP1 deduced amino acid sequence. Phylogenetic tree of Rm4E-BP1 deduced amino acid sequence obtained in this study (marked with a star) and sequences retrieved from NCBI GenBank: BAM28764.1 (*Haemaphysalis longicornis);* EFX81736.1 (*Daphnia pulex*); KRT79235.1 (*Oryctes borbonicus*); KZS19304.1 (*Daphnia magna);* XP_002404524.1 (*Ixodes scapularis);* XP_003740398.1 (*Galendromus occidentalis);* XP_008468645.1 (*Diaphorina citri*); XP_013779553.1 (*Limulus Polyphemus); XP_015792963.1* (*Tetranychus urticae);* XP_015908828.1 (*Parasteatoda tepidariorum);* XP_018027170.1 (*Hyalella Azteca);* NP_477295.1 (*Drosophila melanogaster*); XP_001846418.1 (*Culex quinquefasciatus);* XP_006569762.1 (*Apis mellifera)* and XP_317732.2 (*Anopheles gambiae)*. The phylogenetic relationship was inferred using the Neighbor-joining method. Bootstrap values are shown next to the branches (1000 bootstrap replications).

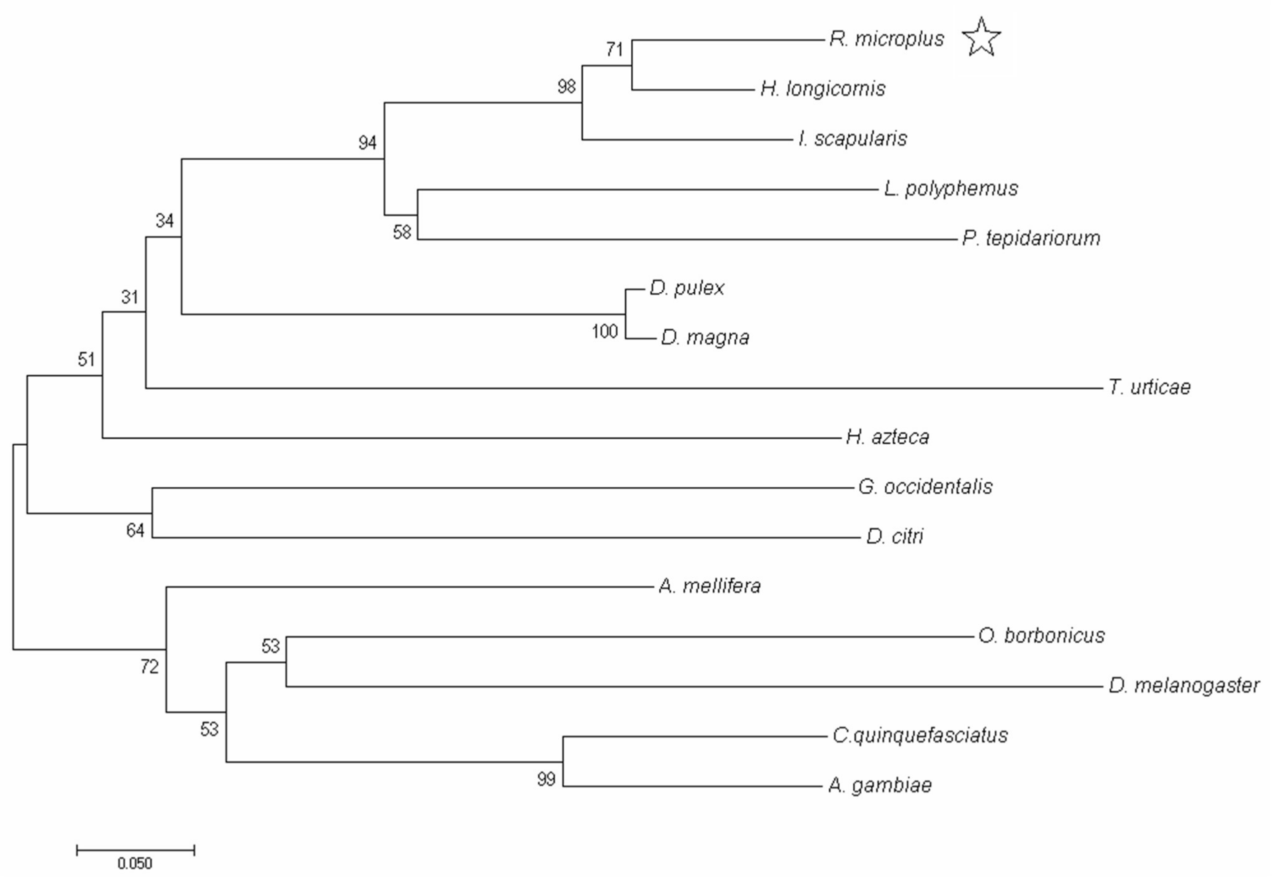

Fig. S3. Confirmation of RNAi-mediated gene silencing for AKT (A), GSK3β (B) and TOR (C) by qRT-PCR in BME26 cells. Cells were plated into wells of 24-well plates, and the final volume of 500 μL was completed with fresh complete medium. Cell plates were incubated at 34°C for a period of 24 h to allow cell adhesion. Subsequently, the culture medium was replaced with 200 μL of fresh medium containing 4 μg of dsRNA and the plate was gently mixed. After 24 h of incubation at 34°C, cells were collected and processed for RNA extraction. RNA from cells treated with dsGFP was used as a control group. All treatments were performed in three independent biological samples and three technical replicates. The plots present mean ± SD from three independent experiments (*= $p<0.05$; **=$p<0.005$; ***=$p<0.0001$).

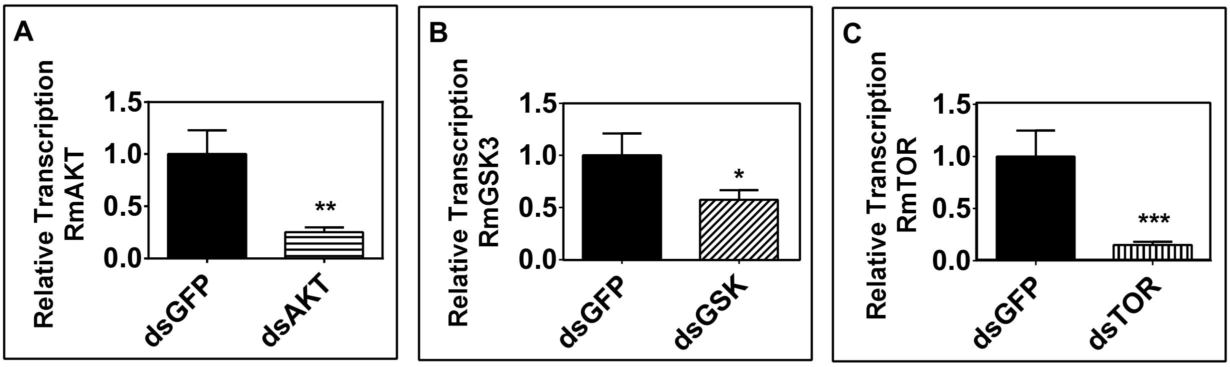

Fig. S4. Transcription of various targets in BME26 cells upon RNAi-mediated gene silencing of three signaling components: RmAKT (A), RmGSK-3β (B) and RmTOR (C). Cells were plated into wells of 24-well plates, and the final volume of 500 μL was completed with fresh complete medium. Cell plates were incubated at 34°C for a period of 24 h to allow cell adhesion. Subsequently, the culture medium was replaced with 200 μL of fresh medium containing 4 μg of dsRNA and the plate was gently mixed. After 24 h of incubation at 34°C, cells were collected and processed for RNA extraction. RNA from cells treated with dsGFP was used as a control group. All treatments were performed in three independent biological samples and three technical replicates.

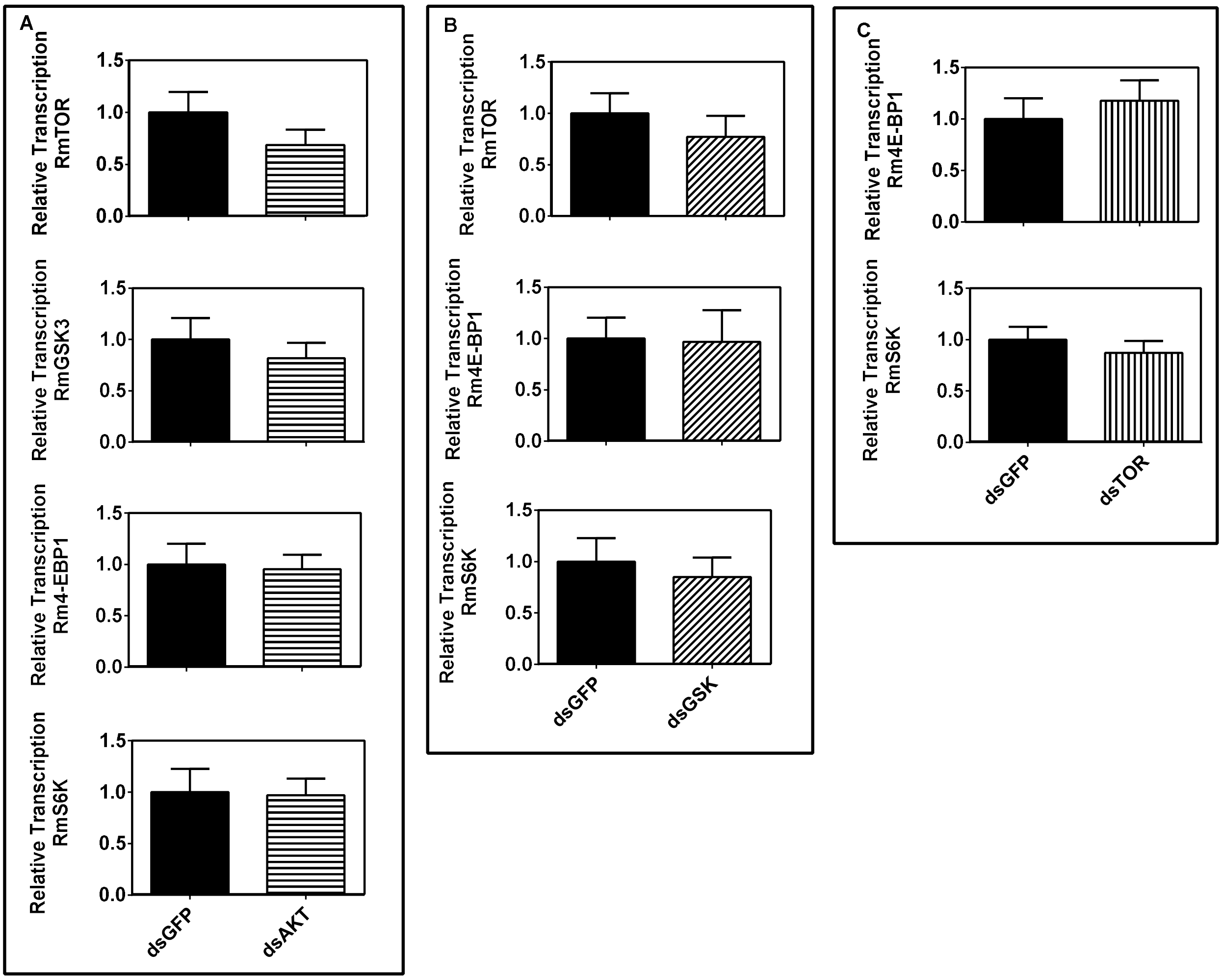

Fig. S5. Confirmation by qRT-PCR of RNAi-mediated TOR silencing in ovaries from partially engorged *Rhipicephalus microplus* female ticks. Females were injected with unrelated dsRNA (4 µg of dsGFP in a maximum volume of 2 µL) or TOR dsRNA (4 µg of dsTOR in a maximum volume of 2 µL). For confirmation of silencing, ovaries from three females for each independent experiment were collected and processed for RNA extraction after 48 h of dsRNA injection. Two independent experiments were performed, with approximately 20 individuals per treatment for each independent experiment. The plot present mean ± SD from two independent experiments (***=p<0.0001).

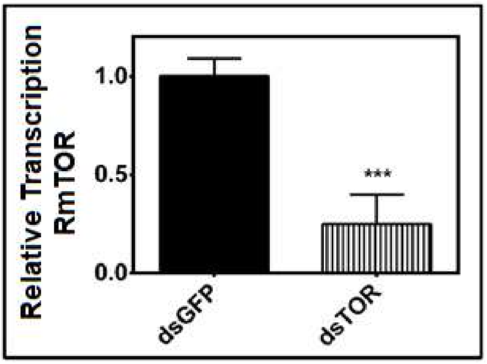

Supplement: Supplementary file 2 [file Data_Sheet_1.PDF]
